# Diversity and Classification of Genetic Variations in Autism Spectrum Disorder

**DOI:** 10.3390/ijms242316768

**Published:** 2023-11-26

**Authors:** Éva Kereszturi

**Affiliations:** Department of Molecular Biology, Semmelweis University, H-1085 Budapest, Hungary; kereszturi.eva@semmelweis.hu

**Keywords:** autism, ASD, genetic variation, SNV, CNV, chromosomal abnormalities, mutation, polymorphism

## Abstract

Autism spectrum disorder (ASD) is a neurodevelopmental condition with symptoms that affect the whole personality and all aspects of life. Although there is a high degree of heterogeneity in both its etiology and its characteristic behavioral patterns, the disorder is well-captured along the autistic triad. Currently, ASD status can be confirmed following an assessment of behavioral features, but there is a growing emphasis on conceptualizing autism as a spectrum, which allows for establishing a diagnosis based on the level of support need, free of discrete categories. Since ASD has a high genetic predominance, the number of genetic variations identified in the background of the condition is increasing exponentially as genetic testing methods are rapidly evolving. However, due to the huge amount of data to be analyzed, grouping the different DNA variations is still challenging. Therefore, in the present review, a multidimensional classification scheme was developed to accommodate most of the currently known genetic variants associated with autism. Genetic variations have been grouped according to six criteria (extent, time of onset, information content, frequency, number of genes involved, inheritance pattern), which are themselves not discrete categories, but form a coherent continuum in line with the autism spectrum approach.

## 1. Introduction

In the early 2000s, the Human Genome Project (HGP) established the first complete genome sequence as part of a broad international collaboration. The knowledge of the three billion base pairs of the human genome, together with the methodological and technical skills accumulated during the project, has clearly set the direction of research for professionals in the field since the turn of the millennium [1]. As in many areas of basic and applied research, the study of the genetic background of a condition has become increasingly important in psychology and psychiatry. Twin studies on autism spectrum disorder have shown that it is one of the most genetically determined neurodevelopmental disorders, with an inheritance rate of between 0.7 and 0.8. It also has a high prevalence, affecting around 1% of the population (see below). It is not surprising therefore, that this developmental disorder, after being first described by Kanner [2] and Asperger [3], very quickly became the focus of interest of clinicians and then of geneticists with the emergence of the theory of genetic determination. Prior to the 2000s and the HGP, mainly the more extensive genomic abnormalities (e.g., chromosomal abnormalities) were studied in relation to ASD. In the years following the HGP, however, there has been increasing interest in exploring the role of smaller, even single-base human variants.

In terms of methodology, psychogenetics has typically investigated the genetic variations of genes selected on the basis of psychological theories and physiological functions as well as their molecular biological effects. However, it soon became clear that a single or even a few genetic variations alone could not explain the vast majority of ASD cases, and the complex, polygenic nature of the developmental disorder, involving hundreds of genes in complex networks, became apparent. If, however, it is indeed the combined effect of many variants of many genes that result in the development of the autistic phenotype, it is legitimate to raise the idea in the psychology, psychiatry, and special education communities that ASD is nothing more than a different organization of the brain from what is currently considered typical. Due to the diversity of the genetic background of autism, research is nowadays shifting toward high-throughput genetic screening tests to understand the mechanisms of ASD at the molecular level, which are highly complex and involve many physiological and biochemical processes.

In addition to reviewing the milestones underlying the genetic determination of ASD, given the complexity of genetic variations in autism and their inconsistent clustering principles in the literature and varying dimensions from publication to publication, the present review aimed to develop a possible classification of DNA-level variations, illustrated with specific examples.

## 2. Genetic Aspects of Autism Spectrum Disorder

### 2.1. Autism Spectrum Disorder

Autism spectrum disorder is a pervasive neurodevelopmental condition with symptoms that affect the whole personality and everyday behavior patterns and persist throughout life. ASD can be considered as a developmental disorder, a specific developmental pathway, but the difficulties encountered are highly dependent on the environment, in addition to the severity of the autism [4]. There is a high degree of heterogeneity in both etiology and characteristic behavioral patterns, but the unity of the disorder is still well-captured along the lines of the autistic triad, or more recently the dyad [5]. Despite the extremely diverse clinical picture, qualitative developmental differences in (i) reciprocal social behavior and (ii) communication (which form a shared category in the dyadic conception) are always present. In addition, (iii) rigid, repetitive patterns of behavior and narrow, stereotyped interests are very common as a third member of the triad. The characteristic symptoms appear early in development, before the age of three. Although not part of the autistic triad, sensitivity to various stimuli (e.g., light, sound, smell, texture, etc.) is frequent (in the dyad concept, this has been placed in the second group of the dyad) as well as an uneven ability profile and insular, outlier abilities (e.g., above-average memory, drawing skills, etc.). The condition can now be interpreted as a multidimensional spectrum, where the specific symptom picture is influenced by several factors. Age, severity of autism, intelligence level, personality, speech production and comprehension, and environmental influences are important factors in the considerable heterogeneity of the clinical picture. The latter include family environment, institutional care, and the extent and quality of support [6].

### 2.2. Epidemiology

The study of the epidemiological aspects of autism spectrum disorder is extremely important for several reasons, as the prevalence of ASD and its alteration influence the planning of the range and availability of care and services for those affected at the societal level. In addition, the prevalence characteristics of a condition may be related to its underlying causes. Accordingly, the temporal and spatially uniform distribution of autism implies a genetic background, as opposed to other conditions that are temporally and geographically uneven and mainly dependent on environmental factors.

#### 2.2.1. Prevalence

**Figure 1:**
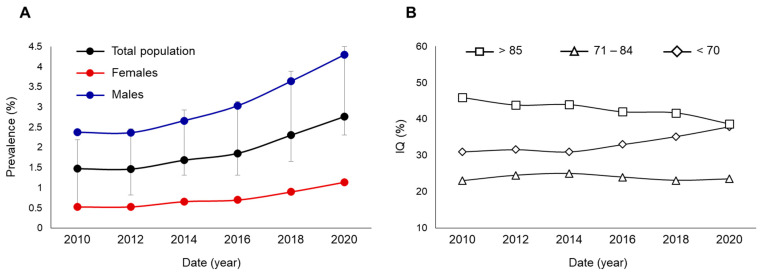
Trends in the prevalence (**A**) and IQ (**B**) of children affected by autism in the United States between 2010 and 2020.

Since the 2000s, data on the prevalence of autism worldwide have shown an increasing trend [7]. While by the turn of the millennium, diagnoses based on Kanner’s description estimated that 4–5 out of 10,000 people were affected, the prevalence of autism began to rise significantly, reaching 1% by 2010, and has continued to increase worldwide over the past 10 years. In an analysis of the geographical distribution, data from the U.S., Europe, Asia, and Africa were combined to find that the prevalence of ASD ranged from 0.2% to 2.5% worldwide [8]. A similar study using post-2012 data estimated an average global prevalence of 1% with a considerably wide range (0.01% to 4.3%) [9]. The staggering increase in the prevalence of autism in the United States since 2010 is also marked in the overall and gender-specific prevalence rates for the eight-year-old child population, which is systematically analyzed and published every two years (Figure 1, [10,11,12,13,14,15]).

Thus, over the last ten years, the prevalence of autism has increased from 1.47% to 2.76%, according to the U.S. public health data. The rate of increase is clear for both sexes (Figure 1A), but there is only a tendency for the IQ distribution of those affected to shift toward the lower range (Figure 1B). Similar trends can be observed both in Europe [16] and on the Asian continent [17], although the data have been less systematically processed thus far. The increase in prevalence can be explained partly by apparent and partly by real case number increases. The apparent increase is due to the broadening of the diagnostic criteria for autism, with a direct consequence of the increasingly thorough recognition of the condition [18]. In addition, there has been a gradual and significant improvement in diagnostic tools and procedures as well as an increase in the knowledge of society regarding autism [19]. The biological explanations for the actual increase are currently unknown, but factors may include selective immigration (e.g., moving into better care settings, the stabilizing effect of cultural distance on the relationship of high-functioning ASD individuals), evolving rehabilitation options, and environmental influences [20]. However, the geographic differences in prevalence, sometimes reaching orders of magnitude, are more likely to reflect the differences in diagnostic protocols and the differences in the level of development of economic and social functioning in different regions of the world, rather than real and significant regional differences [18].

Autism is more common in males, but while published data in the 2000s suggested that 4–5 times more boys than girls were affected, recent studies suggest that the male-to-female ratio is probably closer to 3:1 [21]. It is also noteworthy that at higher IQ levels, the ratio is even more skewed toward males (10–15:1), but at lower IQ levels, it almost evens out (1.5:1) [22]. Recent theories suggest that this is due to ‘female camouflage’, which refers to the ability of high-functioning ASD females to adapt to the rules of mainstream society at the cost of a significant energy investment, better than men and lower-intellectuals [23,24,25,26]. Of course, this comes at a ‘cost’, as non-diagnosis, delayed diagnosis, or misdiagnosis deprives affected females of key support services, which greatly impairs their quality of life, often leading to the development of comorbid mental disorders [27,28,29].

The prevalence of autism among siblings of individuals diagnosed with ASD is much higher than in the general population [30]. On average, it is between 10% and 20%, but the prevalence is influenced by the gender of the person with ASD and the gender of the sibling [31]. Among the siblings of children with autism, the prevalence of subclinical autistic traits that do not reach diagnostic criteria (i.e., the broad autism phenotype) is much higher in neurotypically developing children [32,33].

#### 2.2.2. Heritability

The hereditary nature of autism spectrum disorder is now a clear scientific fact, but there is still some uncertainty regarding its extent. A meta-analysis in 2016 reviewed 6413 sets of monozygotic and dizygotic twins with at least one child diagnosed with ASD. Their results suggest an estimated heritability of 0.64–0.91, which has since been confirmed by others [34], suggesting a currently accepted heritability rate for ASD of 0.7–0.8 [35]. This implies the presence of a similar degree of genetic component as, for example, that of body height [36] or attention deficit hyperactivity disorder (ADHD), which is otherwise comorbid with autism [37].

### 2.3. Etiology

Research into the development of autism spectrum disorder is no longer concerned with specific root causes, but rather with predisposing and protective factors [38]. While the former increases the likelihood of developing the disorder, the latter counteracts it, thus the phenotype is the result of both sets of factors. Since the heritability of ASD is high, but not equal to 1, it is not only the genes and their variations that are responsible for the development of the condition. Therefore, the role of environmental, possibly psychogenic factors in the background of autism cannot be ignored.

#### 2.3.1. Environmental Impacts

The current scientific consensus suggests that the following environmental factors may contribute to the development of autism [39,40,41]: (i) higher parental, especially paternal, age; (ii) adverse prenatal influences (e.g., infections during pregnancy, extreme intrauterine hormonal, especially testosterone effects [42], poor quality of maternal nutrition, maternal substance use and smoking, maternal health, heavy metal pollution or air pollution); (iii) perinatal complications (e.g., umbilical cord abnormalities, meconium amniotic fluid aspiration, low birth weight, etc.) [43]; (*iv*) low socioeconomic status. It is important to emphasize, however, that none of these factors are autism-specific; they increase the susceptibility of many other mental or physical disorders in addition to ASD. Furthermore, some of the environmental factors cannot be sharply separated from genetic effects, as, for example, higher paternal age increases the prevalence of de novo mutations during spermiogenesis [44], and complications during childbirth may also arise due to atypical fetal positioning (e.g., breech presentation, facial positioning, improper fitting in the birth canal), which may also be genetically determined [45].

#### 2.3.2. Genetic Factors

The genetically determined nature of autism spectrum disorder is therefore well-established, but the methodological complexity of assessing the components still makes it challenging to evaluate the accumulated data.

##### Twin Studies

Twin studies of psychiatric disorders such as ASD have employed epidemiological approaches that determine heritability by comparing the concordance rate between monozygotic (MZ) and dizygotic (DZ) twins. Theoretically, MZ twins share 100% of the genetic and environmental background, although this is no longer clear in either case due to epigenetic modifications or de novo mutations [46]. Several such studies have been conducted in the literature with rather heterogeneous results (summarized in Table 1). In a meta-analysis published in 2016, the concordance rate for MZ twin pairs was found to be 98%, while that for DZ twins was 53% [47].

##### Family Studies

The analysis of co-occurrence between siblings of different ages also supports the genetic determination of ASD. This involves examining how often a younger sibling of a child with autism receives a similar diagnosis. In a follow-up study of a relatively large U.S. sample (N = 664), Ozonoff and colleagues found a recurrence risk of 18.7%, suggesting a very strong genetic influence compared to the average prevalence of 1% in the population. Male siblings of children with ASD are three times more likely to have a similar diagnosis than female ones (26.2% vs. 9.1%) [30]. Hansen and colleagues studied the prevalence of autism in an international sample (U.S., Denmark, Sweden, Finland, Australia, and Israel) of more than 60,000 younger siblings and cousins of children diagnosed with ASD and childhood autism. The likelihood of a younger sibling being diagnosed with autism was 8.4 times higher for ASD and 17.4 times higher for a diagnosis of childhood autism. The probability, although smaller (2-fold), was also increased in second-degree relatives [33]. Another study of a large number of cases found an even higher recurrence probability of 30.4 times for a sibling diagnosed with ASD [52]. Although the exact magnitude of the risk varies widely, there is a clear familial cumulative pattern of autism. It is very likely, however, that such family studies have been somewhat underestimated, and the probability of the condition recurring in younger siblings of an older autistic child is presumably higher than that measured, since often after the birth of an affected child, the parents will no longer have more offspring, even if a larger family was previously planned [53].

##### Comorbidities

ASD is associated with hundreds of genetic diseases and conditions, most of which are extremely rare. Approximately 45% of people with autism are intellectually disabled (ID), 50% live with ADHD, and 30% have epilepsy [54]. While AHDH has a very significant genetic component, the same is still only plausible for ID [55] and some types of epilepsy [56]. The prevalence of ASD in genetic syndromes with at least 0.01% frequency is summarized in Table 2.

It is striking that the prevalence of ASD is much higher (up to more than 60 times) than the population average for the listed syndromes. In addition, there are several other very rare genetic disorders associated with autism (Angelman, Joubert, Smith–Lemli–Opitz, and Timothy syndromes, etc. [64]), the prevalence of which is extremely low. To date, approximately 20–35% of individuals diagnosed with ASD have some genetic abnormalities [70], which present a very diverse phenotypic picture, further complicating the possibility of establishing uniform clinical and diagnostic categories. It is noteworthy, that ASD is highly prevalent in certain syndromes compared to the population average, however, among individuals with autism, these syndromes are relatively rarely identified, explaining at most one third of cases [71].

## 3. Categorization of Genetic Variations Associated with Autism along Six Dimensions

The DNA sequence changes underlying autism spectrum disorder can be very diverse. These can range from single nucleotide changes to the appearance of entire extra chromosomes, from rare mutations to very common polymorphisms, from de novo variants to hereditary ones. The picture is further complicated by the blurred boundaries and a lot of overlap between the categories as well as the interpretational differences of modifications affecting the genome [35,70]. The present work attempts to develop and present a transparent categorization system as well as illustrate the significance of a given genetic modification through a couple of autism specific examples.

### 3.1. Categorization by Extent

#### 3.1.1. Chromosomal Abnormalities

The prevalence of autism is significantly increased by certain trisomies. Down syndrome with chromosome 21 trisomy is more than 40 times more likely to develop ASD compared to the population average (Table 2) [58]. Supernumerary sex chromosomes also significantly increase the susceptibility to ASD [72]. On average, the prevalence of autistic traits in people with Klinefelter syndrome is 25% [73,74], and 10–27% of children meet the diagnostic criteria for ASD, a significant increase compared to the population average of 1–2% [74,75]. The prevalence of autism in XXX trisomy is also increased, at around 15% [74,76]. XYY trisomy also predisposes to autism with a high variance; around 30% reach the threshold for clinical diagnosis [77,78]. Interestingly, the only non-lethal whole-chromosome monosomy, Turner syndrome, is not associated with an increased risk of ASD [79].

If a large segment of one chromosome, usually an arm, is lost, the cell is functionally monosomic with respect to the genetic information in that region. In autism, such defects of chromosome 11 are frequently reported in the literature, for example, it is known that loss of a long arm of chromosome 11 (Jacobsen syndrome) is associated with the development of severe autistic symptoms [80]. Nevertheless, Jacobsen syndrome is quite rare, and the prevalence of ASD cannot be accurately established in this population. However, in the case of Cri du chat syndrome with complete absence of the shorter arm of chromosome 5, the prevalence of ASD has increased significantly to almost 40% [81].

The question may arise as to how long a chromosomal abnormality is considered a partial monosomy and when it is considered a deletion, which can be classified more as a structural change. There is no clear consensus on this point. In this work, partial monosomy was considered when one arm of the chromosome was missing in its entirety, while it was classified as a structural aberration when this absence was partial.

Although the structural variations no longer affect the whole chromosome or one of its arms, they are still very extensive, affecting several to hundreds of genes at a time. Their development requires a spontaneous or induced breakage of the chromosome(s) at one or more points. In an unbalanced translocation, the displacement of two chromosome fragments results in the loss of genetic information and phenotypic consequences. The imbalanced variant of the translocation between the short arms of chromosome 8 and chromosome 4 shows familial accumulation and is more common in the ASD population [82,83]. In addition, one-fifth of autism-related cases of Phelan–McDermid syndrome are due to the unbalanced translocation of segments of chromosome 22 and 2 [84]. Prader–Willi and Angelman syndromes are caused by deletion of the long arm of chromosome 15. The incidence of autism in both syndromes is significantly higher than in the control population. While the former is 3–57 times more common [85], the latter has a higher prevalence of more than 30 times for an associated ASD diagnosis [64]. Additionally, other structural anomalies of chromosome 15 such as isodicentric chromosome [86] and uniparental disomy [87] have also been described in the context of autism. Microdeletions are defined as the loss of a relatively small chromosomal fragment, but this does not necessarily imply a favorable outcome. From schizophrenia to cardiac malformations and autism, a number of conditions with a genetic background of microdeletions have been described in the literature (for a summary, see [88]), which highlight several microdeletion hot-spots with an increased frequency of abnormal chromosomal changes. These include the long arms of chromosomes 22, 17, 16, and 7. The vast majority (~80%) of cases of autism-related Phelan–McDermid syndrome are caused by a break at the end of the long arm of chromosome 22 [84]. The same syndrome can also be due to a ring chromosome resulting from the fusion of two broken ends of chromosome 22.

#### 3.1.2. Copy Number Variations

Copy number variations (CNVs) are very frequent, accounting for 5–10% of the human genome [89]. The vast majority (~95%) are common variants that are assumed to be important in the development of genetic diversity [90]. Roughly 1% of them are considered rare variants, which may play a key role in the genetic background of many neuropsychiatric or developmental disorders including autism [91]. The results of a whole genome analysis of a sample of thousands of individuals by Marshall and colleagues clearly showed that all regions, except chromosome Y, were affected [92]. However, several CNVs that have recurred in recent studies clearly highlight some autism-specific chromosomal regions [93] including segments of the long arm of chromosomes 1, 7, 15, and 22, and some areas of the short arm of chromosomes 2 and 16 [93] (Table 3).

As CNVs can result in the simultaneous loss or duplication of up to dozens of genes, the exact connection between autism and these genetic variations as well as their mechanism remains a major challenge. The association between phenotype and changes in the dosage of the affected gene (deletion or duplication) may provide guidance, although the connection is not always predictive. A good example is the microdeletion of the 7q11.23 region in Williams syndrome, which is associated with a high degree of sociability. At the same time, duplication of the same region is likely to result in autism and communication deficits [97,98,99]. This suggests that the region may have dose-dependent opposite effects in the phenotype. However, individuals with microdeletion, similar to those with ASD, have been found to have basic social skills deficiencies [100]. This is not surprising given the complex nature of the different social functions and behaviors, so the relationship between the genotype and phenotype is likely to be even more nuanced.

Interestingly, the deletion and duplication of another CNV affecting the 16p11.2 locus were both equally associated with autism, but while deletion was correlated with macroencephaly and obesity [101], duplication was phenotypically characterized by microencephaly and impaired growth [102]. An attempt to explain this paradox relationship was made by Deshpande and colleagues [103]. Fibroblasts from individuals with deletion or duplication of the 16p11.2 region were used to generate pluripotent stem cell cultures and differentiate the cells in the neural direction. Neurons carrying the deletion had larger cell bodies and longer dendrites than those carrying the duplication. Despite the significant phenotypic difference, however, neurons with either deletion or duplication showed similar levels of reduced synaptic activity, offering very elegant evidence for a correlation between clinical phenotype, cellular processes, and genetic background.

#### 3.1.3. Single Nucleotide Variations

Single nucleotide variations (SNVs) compose about 0.1% of the human genome, the majority of which are common variants and, like CNVs, are a major source of genetic diversity. Due to their extent, SNVs can usually affect only one gene. On the one hand, this may facilitate the identification of their role in a given phenotype, in this case autism, but on the other hand, the complex nature of ASD means that the small effect of a single gene can easily be ‘lost’ in the interpretation of the different neurodevelopmental background of an intricate interplay of tens or even hundreds of genes. Today, a very large number of genes and their SNVs are associated with autism in the literature, and the list is expected to grow with the routine application of next-generation whole-genome sequencing. Processing this dynamically growing amount of data is largely facilitated by a number of searchable databases of SNVs and genes associated with ASD. The most recent online databases available without restrictions are listed in Table 4.

However, the results of the various high-throughput studies and the growing number of summaries written on the topic [35,93,104,105] are still only the beginning of the journey, as it is very common that the list of SNVs and genes associated with ASD from the various works is not or only limitedly overlapping. To resolve these discrepancies, further, largely functional studies and advanced bioinformatical methods will be needed. Despite the contradictory results, three main functional gene clusters seem to emerge in relation to ASD. In addition to a group of genes that are key in the regulation of gene expression and neuronal communication [106], genetic information that controls the organization of the cell scaffold appears to be crucial [104]. A striking example of the latter is the much-studied three isoforms of the *SHANK* gene, which encode post-synaptic scaffolding proteins of glutaminergic synapses in the central nervous system. Many of their SNVs have been consistently shown to be associated with the development of autism [107,108]. In addition, the role of the *NLGN3* and *NLGN4* genes, which encode neuronal cell surface proteins and are thought to be important in regulating intercellular communication, in the etiology of ASD is also clear, and their mutations have been widely identified and studied [109,110].

### 3.2. Categorization by Time of Onset

#### 3.2.1. De Novo Variations

A de novo variation is the result of a mutation in the parental gametes or a genetic modification during embryogenesis that arises for the first time in the individual. Genetic alterations during embryogenesis or later in development result in mosaicism (i.e., only the progeny of the cell that has undergone the genetic change carry the de novo variant). Such genetic variation can occur at the level of chromosomes, CNVs, and SNVs. Although it is not yet entirely clear what proportion of ASD cases involve de novo events, the prevalence of somatic variations in autism has recently been estimated to be between 5 and 7.5% [111,112]. Most of the autism-related chromosomal abnormalities discussed in Section 3.1.1. fall into this category. Rare CNVs (see Section 3.1.2) also often develop during de novo events [96], with an increased incidence of 3–10 times among individuals diagnosed with ASD [113].

#### 3.2.2. Inherited Variations

Hereditary variations can be found in the parental genome. They can affect large gene regions or only a single nucleotide. In general, due to the complexity of the genetic background of autism spectrum disorder, recessively inherited (see also Section 3.6.) genetic alterations are the primary focus of research [114,115]. Although their significance has been described for more than 30 years [116] and demonstrated in a number of ways [117], the extensive study of heritable variants has been somewhat overshadowed by the current interest in de novo variation. In 2013, Lim and colleagues concluded that in about 5% of cases, ASD is caused by inherited variants that result in the loss of function of a particular gene, of which 3% are autosomal and 2% are X-linked [118].

### 3.3. Categorization by Information Content

Given the complex genetic background of ASD, it is not surprising that an extremely large number of autism-related genetic variants affecting the coding region have been described, so this review can only highlight the main trends.

#### 3.3.1. Coding Region Variations

The three billion base pairs of the human genome encode only 20–25 thousand genes, which are translated into protein via mRNA. Genetic variations affecting exons therefore have a high probability of being reflected in the structure of the protein, altering its function. In most cases, this change affects the protein negatively, causing a loss of function and disrupting a very complex and precisely engineered system. Less frequently, variations in the coding region can create a protein that works more efficiently or has a new function (gain-of-function variations). Larger, deletional CNVs tend to corrupt genes, which can often be partially or completely lost. Their duplications, however, can lead to a stronger gene expression, which of course is not necessarily beneficial for the organism. The effect of missense SNVs can range from undetectable to gain-of-function or to complete loss-of-function. Longer or shorter insertions or deletions may also show a variable phenotypic picture depending on the number and position of nucleotides being excised or incorporated. A large-scale study sequencing the whole genomes of more than 100,000 individuals concluded that rare loss-of-function variants in coding regions significantly increased the risk of autism, among several other neurological conditions [119].

#### 3.3.2. Intron Variations

Although intron variations do not appear in the protein structure, specific regions of the introns are essential for mature mRNA function. During the splicing process, the regulatory role of certain intron sequences is essential, so variations in these sequences may already clearly contribute to the phenotype, as has been shown in the case of autism spectrum disorder [120]. It has been raised that in this case, the intron region in question could be considered a regulatory region (see Section 3.3.3). Further complicating the picture, an intron of one gene may be a regulatory region for another one. In addition, in the process of alternative splicing, where a variable number of exons are deleted alongside introns, these intron regions also have a gene- and tissue-specific regulatory role. Given the complexity of the human genome, the picture is therefore complex and the boundaries between categories are not sharp.

#### 3.3.3. Regulatory Region Variations

Regulatory regions ultimately affect the amount of protein produced from a gene in a given cell under given conditions. While the promoter regulates gene expression, the 3′ region affects the stability of the mRNA produced and the process of translation. However, in addition to studying genetic variation in regulatory regions [121], there is increasing interest in comparing mRNA and protein levels that are also affected by genetic modification in samples from individuals with autism and controls. Comparisons of samples from different tissues of healthy and ASD individuals have shown differences in the expression patterns of several genes such as *FOXP1*, *MAL*, and *C11orf30* [122,123,124,125]. This is further supported by the fact that several variants of these genes have also been associated with language development delay and autism [126,127].

#### 3.3.4. Non-Coding Region Variations

Only 1.5% of the human genome codes for proteins. Currently known regulatory regions account for a further 1.5%. A part of the remaining 97% encodes other RNAs (tRNA, rRNA, microRNA, lncRNA), while the function of other parts is still unknown. It is understandable that at the beginning of genetic studies and even today, this large DNA pool has mostly been outside the focus of autism research. Nevertheless, data on the genetic variation of these regions are steadily accumulating with the results of high-throughput whole genome sequencing [128,129]. However, identifying genetic variants associated with autism is only the easy part of the task, as the lack of known function of the DNA sequence makes it challenging to provide a causal explanation. A further complication is that the average effect of these non-coding regions on the development of autism is likely to be relatively small [130].

### 3.4. Categorization by Frequency

The average difference of 0.1–0.5% between the genomes of two unrelated individuals is the sum of rare and common variants. The frequency of rare variants or mutations in the population is below 1%, correspondingly, the frequency of common variants or polymorphisms is above 1%. Other definitions set this threshold as 5%, so the frequency range between 1 and 5% is considered a grey zone.

#### 3.4.1. Rare Variations

In general, mutations tend to, but not exclusively, affect the coding region or the entire gene and have a drastic effect on the expressed protein. As a result, a larger proportion of them is created through de novo events. Chromosomal abnormalities are understandably clearly rare variants, but they are relatively common in autism (see Section 3.1.1.). However, only about 1–4% of CNVs are classified as rare variations. Analyzing the genetic samples of more than a thousand ASD and neurotypical individuals, Pinto and colleagues found a significantly higher prevalence of rare CNVs in autism compared to the control population (7.6% vs. 4.5%) [131,132]. According to the current point of view, rare genetic variations are responsible for only 1–5% of ASD cases, which usually affect genes involving synaptic connections (such as the neuroligin gene family, the scaffolding protein family, or the neurexin gene family) [35].

#### 3.4.2. Common Variations

Polymorphisms are mainly found in regulatory or non-coding regions, with no or mild functional consequences. They largely make up the genetic complexity of polygenic traits, and hence of autism, involving many variations in many genes. Common variants may underlie around 40–60% of ASD cases [133,134]. Two opposing models are known for the frequency of genome variation associated with ASD. One model emphasizes the role of rare variants with high phenotypic impact [124], while the other model suggests that common variants contribute at least 50% to the high heritability of ASD [133]. The latter suggests that these common variants can easily persist in the population due to their small phenotypic effect, in contrast to rare variants with robust phenotypic consequences and high selection pressure [90].

### 3.5. Categorization by Complexity

#### 3.5.1. Monogenic Factors

A trait can be considered monogenic if a single change at the DNA level or one or more genetic modifications in a single gene is sufficient for its development, which is obligate regardless of environmental influences, and is merely the consequence of a change in the genetic information. These conditions usually form discrete categories, with no transition between them. Section 3.1.1. offers several examples of monogenic conditions in connection with autism, reiterating that the mentioned genetic abnormalities are monogenic for the developing syndromes and not for ASD, with a clear contribution to the genetic background of the latter [135].

#### 3.5.2. Polygenic Factors

The development of polygenic traits, conditions, and diseases involves many variations of many genes in combination with environmental influences. As a consequence, they usually do not create discrete categories, but a diverse phenotypic continuum moving across a broader spectrum, depending on the number and quality of the genes involved and the environmental influences. Despite the fact that many types of genetic predisposition have been successfully identified and characterized, currently no single variant, gene, or polygenic score has a high predictive value in the diagnosis of ASD. Even CNVs with a high effect size for ASD present with variable psychiatric characteristic traits, and risk may be attributed to a combination of rare and common variants [136].

### 3.6. Categorization by Inheritance

For many of the genetic variations associated with ASD, the exact pattern of inheritance is still unknown, but where it is, virtually all known possibilities can occur. The present work does not aim to describe all possibilities, but merely to illustrate, through a few examples, another segment of the extremely complex genetic components of autism.

In tuberous sclerosis with autosomal dominant inheritance, the prevalence of autism is quite high, around 25–50% (see Table 2). The development of the disease is due to loss-of-function mutations in two genes involved in the regulation of key cellular mechanisms (proliferation, protein and lipid synthesis, apoptosis), *TSC1* and *TSC2*. Thus, in addition to tumor lesions affecting virtually all organs, ASD symptoms are also very common [137].

Autism-related Smith–Lemli–Opitz syndrome, an autosomal recessive inheritance disorder, is caused by gene defects in a key enzyme of cholesterol metabolism (*DHCR7*). Although the disorder itself is quite rare, the phenotypic picture of the syndrome is closely associated with autistic behavioral features [64,138].

Fragile X syndrome and Rett syndrome are characterized by dominant inheritance linked to the X chromosome. While the former is the most common cause of mental retardation in men, the latter mainly affects women. Both disorders have a relatively high prevalence of autism (30–60%, see Table 2) [64,67]. In Fragile X syndrome, a high number of trinucleotide repeats (CGGs) of the *FMR1* gene on the X chromosome leads to gene silencing and thus a reduction in the amount of protein produced [139]. While the normal number of repeats is below 30, affected individuals have 200–2000 repeats. Mutations in the *MECP2* gene, located at the end of the longer arm of the chromosome X (Xq28), are involved in the development of Rett syndrome. *MECP2* is a transcriptional regulator expressed throughout the body that regulates the function of several other genes, and therefore the full mechanism of disease development is still unclear [140]. The mutations themselves are de novo in the vast majority of cases, but can arise in the maternal germline, which thus exhibit a classical X-linked dominant inheritance pattern.

## 4. Discussion

Over the past two decades, significant progress has been made in understanding the genetic components of autism spectrum disorder. Large sample size studies and technological and bioinformatical innovations have enabled the identification of many common genetic predisposing factors and detailed autism-specific analysis of the human genome. From monogenic and chromosomal abnormalities, which can explain only a very small proportion of ASD cases, the focus has moved on to the study of multicomponent, complex genetic determinants. Moreover, in addition to the importance of rare variations, which is not disputed, there is a growing emphasis on the role of common variants in autism [70].

However, the literature on the genetic components of autism categorizes changes in DNA in very different ways. Often, merely one aspect is discussed (e.g., only CNVs, only de novo variants) or only a few dimensions are highlighted. The principles for grouping genetic variations also vary from publication to publication, making it very difficult to compare them and to form a coherent picture. This review has attempted to classify the genetic variations involved in autism into six categories based on their extent, time of onset, localization inside the gene or genome (i.e., protein coding vs. non-coding), frequency of variation, inheritance pattern, and the number of genes involved in a given individual, thus covering the full spectrum of variations in DNA along several dimensions.

**Figure 2:**
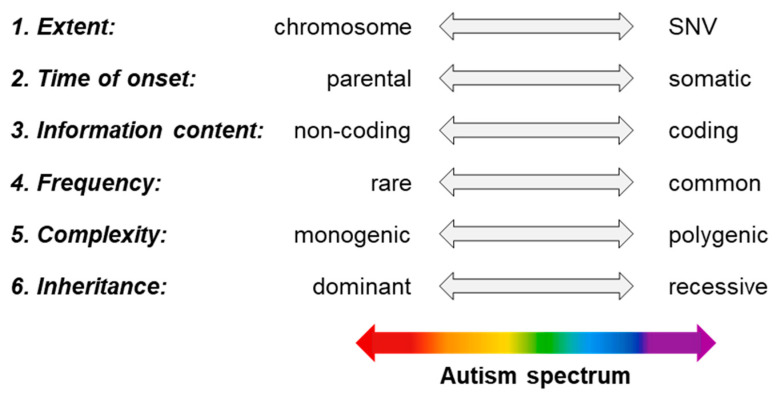
Dimensions of the genetic components of ASD.

The picture is further complicated by the fact that a given genetic variation can often belong to more than one of the dimensions discussed in Section 2 and illustrated in Figure 2. In Phelan–McDermid syndrome, the prevalence of autism is greater than 70% and can be caused by multiple defects on chromosome 22 [141]. Phenotypically similar consequences are also associated with the translocation or deletion of the longer arm of chromosome 22 or mutations affecting one or a few bases of genes in this region (see Figure 2, dimension of extent). In addition, these alterations may be newly induced or even inherited (see Figure 2, dimension of onset).

It is also noteworthy that each of the six dimensions summarized in Figure 2 can be considered a spectrum itself, with the number of transitions between the two endpoints (shown in the figure) being virtually infinite. Thus, it is easy to conclude that the genetic predisposing factors of individuals with autism can be located in a very diverse pattern along the spectrum of the six dimensions, contributing in many different ways and degrees to the autism phenotype. Although ASD is largely a genetic condition, we cannot overlook the variability in neurodevelopment, cognition, and environmental influences, which together make the interpretation of the autism spectrum a complex and challenging scientific and professional endeavor.

Currently, the bottleneck in autism research is no longer the lack of eligible subjects, the complexity of genetic analysis, or the inaccuracy of diagnostic systems, but the evaluation of the accumulated data and the definition of interpretative frameworks. Recent research trends represent an interdisciplinary systems biology and network approach. Nowadays, there is a growing number of consortia studies in this field, where representatives from several disciplines are working together to develop a complex genetic model of autism [104,142,143].

It is undoubtable that the translation of rapidly growing genetic knowledge into clinical, pedagogical, and special education practice is a rather slow process. A question has arisen as to whether and how the person with autism and their family will benefit from the genetic knowledge detailed in this review. The current view is that, although it is now feasible, genetic variants associated with autism are not screened for during pregnancy, as the link between a particular genetic condition and autism is still unclear. There is no guidance on what to do when a variation closely associated with autism is detected in a fetal sample. Today, since it is still only possible to identify genetic constellations that predispose to autism, but not necessarily cause it, ethical questions have been rightly raised in connection with this type of testing. However, there is also a strong argument for pre- and perinatal genetic screening, since if parents are aware of the higher likelihood of autism in the newborn child, intervention as early as possible can give them the best possible outcome.

Genetic testing following a diagnosis of autism can have a number of benefits, as it can provide families with a reassuring explanation of the cause of ASD, help them accept the condition, and avoid self-blame. It can also help people with similar genetic alterations and their families to join a support group. Advanced and detailed genetic testing can also help to identify the presence of other genetically determined conditions (e.g., cardiac malformations, tumors) that may require medical intervention and may go unnoticed in the shadow of autism. Finally, the long-term benefits must be taken into account, as the growing pool of autism-specific genetic information will have an impact on the well-being not only of children currently living with autism, but also of children born with the condition in the future. Overall, the genetic panels developed for screening ASD associated variations cannot yet provide definitive information on whether a person has autism (i.e., they cannot be used for diagnostic purposes). However, they offer at least partial answers to the cause of the condition [144]. Finally, scientific efforts to find a cure for autism, mainly in monogenic cases, using a gene therapy approach, should also be mentioned [135]. However, this raises ethical issues, together with the limitations of technical implementation, and is currently not a reality in clinical practice [145,146].

As researchers gain a deeper understanding of the genetic background of autism, they may be able to assign developmental pathways specific to a genetic pattern, which may allow for the development of tailored, effective interventions [143].

## 5. Conclusions

With the opportunities offered by technological advances, it is now possible to sequence whole genomes in a matter of days, allowing comparative analyses to be carried out. Larger studies often compare the genomes of thousands or even tens of thousands of autistic and typically developing individuals. This has, of course, not only brought technical innovations in molecular biology techniques, but has also led to dramatic advances in comparative analysis, statistics, and, above all, bioinformatics. Alongside genome sequencing, exome and RNA sequencing, which allow for qualitative and quantitative analysis of the ‘active’ part of the genetic material that is being transcribed, are becoming increasingly common. All this can now be carried out from a single cell and without invasive sampling procedures. It should not be overlooked, however, that currently ASD patients are diagnosed and categorized on the basis of behavioral characteristics, the criteria for which may vary between clinical trials and thus affect the interpretation of the results obtained, and it is therefore not surprising that heterogeneous genetic results are reported in a heterogeneous patient population.

In recent years, a huge amount of genetic information has accumulated—and is still accumulating—in the field of autism research, which may legitimately raise the question of whether we are any closer to understanding the etiology of ASD. The answer is not obvious, despite the huge amount of data and work that has been conducted. It can be stated with certainty, thanks to ever-evolving diagnostic tools and newer and newer molecular biology and bioinformatics techniques, that considerably more is known about the molecular genetic background of this condition than was earlier the case, but it is still far from being fully understood. Similarly, with a few exceptions, the possibility of reliable genetic screening or even personalized treatment at the genetic level—if this is even necessary—is still a long way off. Only the details are known, and further network-based research would be needed to discover systems in the dataset that clearly identify the key gene clusters underlying ASD.

According to the current state-of-the-art understanding of autism, ASD is a spectrum condition with no distinct categories. However, this approach is not always useful in clinical and genetic research, which often requires grouping subjects according to certain criteria (e.g., IQ) to achieve greater homogeneity. Ultimately, a clearer diagnostic categorization of ASD will lead to clearer genetic correlations, and clearer genetic definitions would be of great help in autism identification and diagnosis.

## Figures and Tables

**Table 1:** Concordance in mono- and dizygotic twin pairs affected by autism.

Case Number (N)	Gender	Concordance (%)		References
		MZ	DZ	
80	Male	58	21	[48]
62	Female	60	27	[48]
14,794	Mixed	88	43	[49]
554	Mixed	88	31	[50]
74	Mixed	80	14	[51]

MZ: monozygotic twins; DZ: dizygotic twins.

**Table 2:** Prevalence of ASD for genetic syndromes with a prevalence of at least 0.01%.

Genetic Syndrome	Frequency in Population	Prevalence of ASD (%)	Genes/Regions Affected	References
Klinefelter~	1:650 (m)	6	Extra X chr.	[57]
Down~	1:700	42	21 chr. trisomy	[58]
Dup15q~	1:5000	80	Extra copy of 15q11.2-q13.1	[59]
Digeorge~	1:4000	23–41	Deletion of 22q11.2 region	[60,61,62]
Williams–Beuren~	1:7500	19	Duplication of 7q11.23	[63]
CHARGE~	1:10000	50	*CHD7*	[64]
Tuberous sclerosis	1:6000	26–50	*TSC1, TSC2*	[64,65,66]
Rett~	1:1000 (f)	60	*MECP2*	[64]
Fragile X~	1:4000 (m); 1:8000 (f)	30–50	*FMR1*	[64,67]
Neurofibromatosis	1:3000	18–40	*NF1*	[64,68]
Noonan~	1:2000	15	*PTPN11, SOS1, RAF1, RIT1*	[64,69]

(m): Males; (f): Females.

**Table 3:** The most common CNVs in autism.

Genomic Region	CNV	Frequency in ASD (%)	References
1q21.1	Deletion	0.2	[93,94,95,96]
1q21.1	Duplication	0.2	
2p16.3	Deletion	n.a.	
7q11.23	Duplication	0.2	
15q11.2	Deletion	0.5	
15q13.3	Deletion	0.5	
15q11q13	Duplication	0.5	
16p11.2	Deletion	0.8	
16p11.2	Duplication	0.8	
22q11.21	Duplication	0.5	
22q11.2 distal	Deletion	0.5	
22q13.33	Duplication	0.5	
1q21.1	Deletion	0.2	

n.a.: Not available.

**Table 4:** Databases of the human genetic variations associated with autism.

ASD-Spec. Genetic Databases	Website
VariCarta	https://varicarta.msl.ubc.ca/index (accessed on 12 April 2023)
SFARIgene	https://gene.sfari.org/ (accessed on 12 April 2023)
AutDB	http://autism.mindspec.org/autdb/Welcome.do (accessed on 12 April 2023)
ClinVar	https://www.ncbi.nlm.nih.gov/clinvar/ (accessed on 12 April 2023)
Autism Genetic Database	http://autism-genetic-db.net/ (accessed on 12 April 2023)

## Data Availability

All data are available in the main text. This study does not include no data deposited at external repositories.
